# An Extensive Calcifying Epithelial Odontogenic Tumor: Multidisciplinary Diagnostic Approach

**DOI:** 10.1155/crid/6290361

**Published:** 2026-10-22

**Authors:** Manika Shrestha, Prabhat Chandra Thakur, Dipshikha Bajracharya

**Affiliations:** ^1^ Department of Oral and Maxillofacial Pathology Kantipur Dental College Teaching Hospital and Research Center Kathmandu Nepal; ^2^ Department of Head & Neck Oncology Nepal Cancer Hospital and Research Center Lalitpur Nepal

**Keywords:** calcifying epithelial odontogenic tumor, case report, odontogenic tumor, oral squamous cell carcinoma

## Abstract

A calcifying epithelial odontogenic tumor (CEOT), also known as a Pindborg tumor, is a rare benign odontogenic neoplasm with variable biological behavior and potential for locally aggressive growth. Its clinical, radiological, and histopathological features may mimic malignancy, creating diagnostic challenges. We report the subsequent clinical course and definitive management of an extensive CEOT in a 34‐year‐old woman whose lesion had previously been reported as CEOT. Approximately 8 years later, she presented with persistent nonhealing ulcer of the right buccal mucosa and progressive swelling of the right posterior mandible. An incisional biopsy performed at another institution was diagnosed as oral squamous cell carcinoma (OSCC). The patient was subsequently referred to specialized cancer hospital for further evaluation. Repeat histopathological examination revealed anastomosing islands and well‐defined nests of polyhedral tumor cells with eosinophilic cytoplasm and centrally located round nuclei. Basophilic calcifications and homogenous eosinophilic material were also observed, supporting the diagnosis of CEOT. Considering the aggressive clinical presentation and extensive involvement, segmental resection of the mandible followed by reconstruction was performed. The case emphasizes the importance of careful clinicoradiographic and histopathological correlation in establishing an accurate diagnosis and guiding appropriate management of CEOT.

## 1. Introduction

The term “Pindborg tumor” was coined in honor of Dr. J. J. Pindborg of Denmark, who first described this rare neoplasm in 1955. It is also referred to as calcifying epithelial odontogenic tumor (CEOT), with a reported incidence of less than 1% [1]. CEOT is an odontogenic neoplasm composed entirely of epithelial tissue; however, its histogenesis remains uncertain. Some authors suggest that the tumor originates from the stratum intermedium of the enamel organ during tooth development, whereas others propose that it arises from remnants of the primitive dental lamina present in the early stages of odontogenesis [2, 3]. According to the 5^th^ edition of WHO Classification of Head and Neck Tumors (2022), three histopathological subtypes of CEOT are recognized: clear cell CEOT, cystic/microcystic CEOT, and noncalcifying/Langerhans cell‐rich CEOT [4]. CEOT may exhibit diverse clinical, radiographic, and histological features that can mimic malignant neoplasm. Prominent polyhedral epithelial cells with abundant eosinophilic cytoplasm and marked pleomorphism may impart a squamoid appearance, resembling squamous cell carcinoma [5]. The clear cell variant may mimic other malignant clear‐cell neoplasms, including clear cell odontogenic carcinoma, central mucoepidermoid carcinoma, and metastatic renal cell carcinoma [6, 7]. Recognition of characteristic amyloid‐like deposits and Liesegang‐ring calcifications is important for distinguishing CEOT from these entities. Other benign odontogenic lesions that may mimics CEOT includes adenomatoid odontogenic tumor (AOT), calcifying odontogenic cyst (COC), ameloblastic fibro‐odontoma (AFO), and odontoma [3, 8].

The present case is particularly noteworthy because it represents the subsequent clinical course of CEOT previously reported by Baral et al. in 2020 [2]. In the earlier report, the patient presented with a right posterior mandibular swelling, and histopathological examination demonstrated sheets of polyhedral epithelial cells, homogeneous eosinophilic materials, and areas of calcification leading to a diagnosis of CEOT.

The present report describes the subsequent progression and extensive presentation of CEOT in a 34‐year‐old woman with an 8‐year history of nonhealing ulcer of the right buccal mucosa, clinically mimicking oral carcinoma. The diagnostic challenge was further heightened by the lesion′s extensive, locally aggressive behavior and its interim diagnosis as oral squamous cell carcinoma (OSCC). Repeat clinicoradiographic and histopathological evaluation established the diagnosis of CEOT. Thus, this report provides additional clinical, diagnostic, therapeutic, and follow‐up information that expands upon the previously reported presentation of the lesion.

## 2. Case Presentation

A 34‐year‐old woman presented to the Head and Neck Oncology Unit with a chief complaint of a nonhealing ulcer of the right buccal mucosa persisting for 8 years, along with swelling over the right angle of mandible for the preceding 3 months. The patient had a history of previously reported CEOT involving the right posterior mandible. The earlier case was published by Baral et al. in 2020, when patient was 26 years old [2]. At that time, she had presented with a right posterior mandibular swelling of approximately 1.5‐year duration, associated with intermittent dull aching pain and mobility of mandibular second molar. Clinical, radiographic, and histopathological examination led to a diagnosis of CEOT, and segmental resection of the mandible followed by reconstruction was planned. However, definitive treatment was not documented in the previous publication [2].

Following the initial diagnosis, the patient did not undergo documented definitive treatment and subsequently presented to another hospital because of persistence and progression of the lesion. An incisional biopsy was performed at that institution, and the lesion was diagnosed as OSCC by a general pathologist. In view of the clinical extent of the lesion and the diagnosis of malignancy, the patient was subsequently referred to a specialized cancer hospital for further evaluation and management. The initial diagnostic discrepancy may have resulted from the limited nature of the incisional biopsy and its inability to adequately represent the underlying lesion.

The swelling was associated with intermittent dull aching pain and Grade I trismus. Extraoral examination revealed a diffuse swelling measuring approximately 8 × 5 cm over the right angle of the mandible and submandibular region, resulting in noticeable facial asymmetry. On palpation, the swelling was firm, nonfluctuant, and immobile. The overlying skin was erythematous and ulcerated (Figure 1). In addition, the right submandibular lymph nodes were palpable and tender. Intraoral examination revealed an ulceroproliferative lesion involving the right posterior mandibular alveolar ridge, extending from the distal aspect of second molar to the posterior part of retromolar trigone (Figure 2). The lesion resulted in obliteration of the buccal and lingual vestibule, with consequent displacement of the right mandibular first molar.

**Figure 1:**
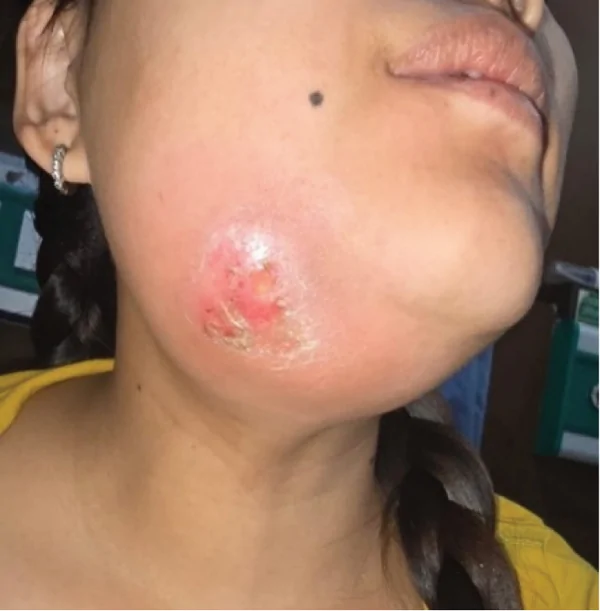
Extraoral swelling in the right lower back region.

**Figure 2:**
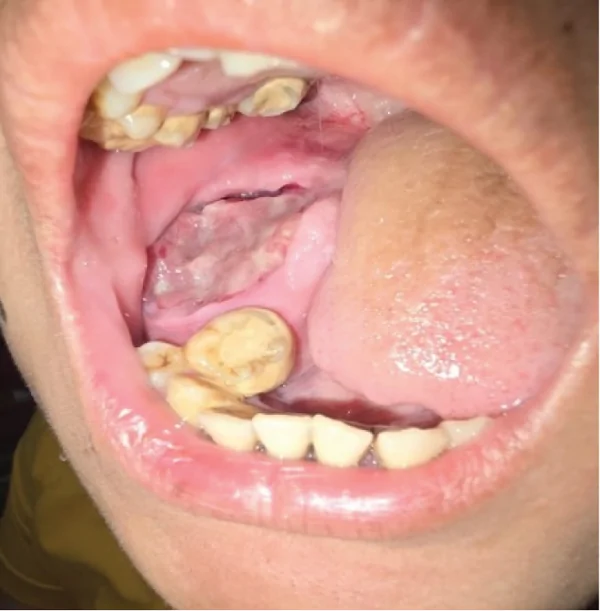
Intraoral picture revealed an ulceroproliferative lesion.

Contrast‐enhanced computed tomography (CECT) of the craniofacial region revealed a large, ill‐defined lytic lesion involving the right body and ramus of the mandible, measuring approximately 65 × 48 mm. The lesion demonstrated a heterogeneously enhancing solid component with coarse internal calcifications and central nonenhancing areas (Figure 3). Expansion and cortical breach were evident, with extension of the lesion into the oral cavity and abutment of the right lateral border of the tongue. A few enlarged right level Ia and Ib cervical lymph nodes were noted, the largest measuring approximately 14 × 12 mm. Three‐dimensional volume‐rendered CT reconstruction of the craniofacial skeleton was also performed (Figure 4a,b).

**Figure 3:**
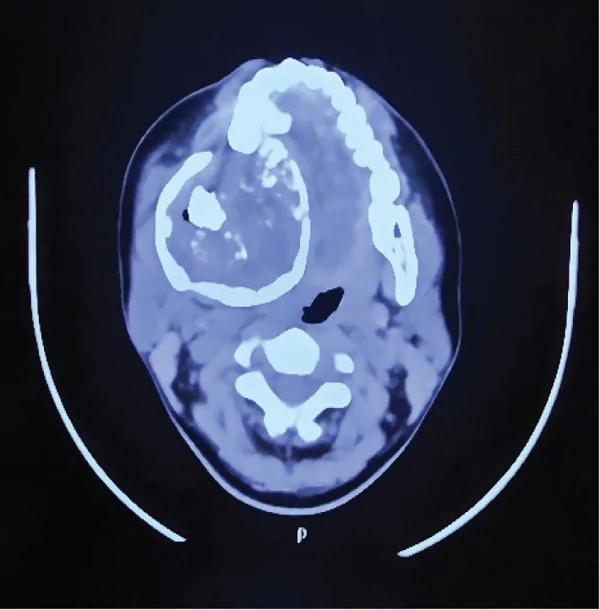
CECT of the craniofacial region showing coarse internal calcifications and central nonenhancing areas.

**Figure 4:**
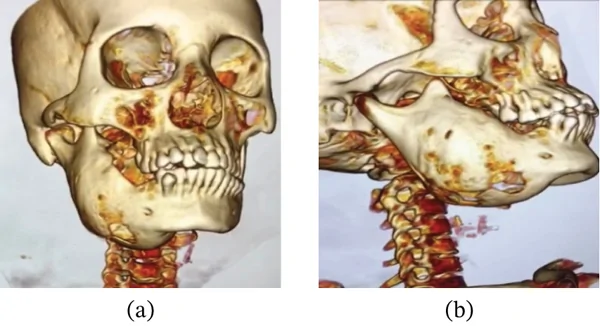
Three‐dimensional volume‐rendered CT reconstruction of the craniofacial skeleton. (a) Frontal view and (b) oblique view.

In view of the extensive clinical and radiographic findings and the discrepancy between the previous diagnosis of CEOT and the subsequent diagnosis of OSCC, a repeat incisional biopsy was performed. Gross examination revealed two pieces of soft to firm, whitish tissue measuring together approximately 1 × 1 × 0.4 cm. Histopathological examination demonstrated a tumor arranged in anastomosing islands and well‐defined nests composed of closely packed sheets of polyhedral epithelial cells. The tumor cells exhibited well‐defined borders, eosinophilic cytoplasm, and centrally located round nucleus. No significant cellular atypia, mitotic figures, necrosis, or keratin pearl formation was identified in the section examined. Areas of basophilic calcification (Liesegang ring) and homogenous eosinophilic material suggestive of amyloid‐like deposition were also observed (Figure 5a,b). Based on these findings and correlation with the clinical and radiographic features, a diagnosis of CEOT was established.

**Figure 5:**
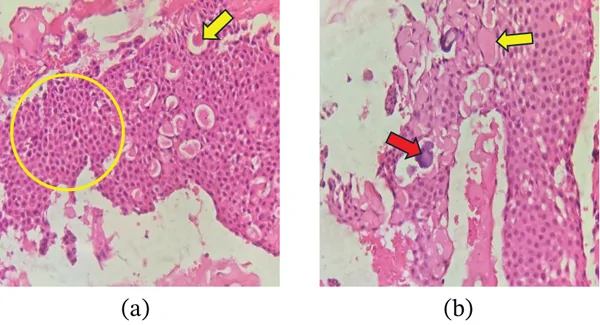
Photomicrographs showing sheets of closely packed epithelial cells with prominent intercellular bridges (circle), amyloid‐like deposition (yellow arrow), and Liesegang ring calcification (red arrow) in (a, b) H&E‐stained sections, 40× magnification.

Considering the extensive and locally aggressive nature of the lesion, wide surgical excision was performed through an extended submandibular approach with a vertical angle split. Adequate peripheral skin margins were included around the area of cutaneous discharge, along with an extended supraomohyoid neck dissection encompassing level I–III (Figure 6a,b). Reconstruction was performed using a left free fibula flap with a 12 × 6 cm skin paddle. The fibula was contoured into three osteotomy segments and fixed using a reconstruction plate and screws. Microvascular anastomosis was established between the fibular artery and peroneal artery and between the common facial vein with vena comitans. The donor‐site defect was reconstructed using a split‐thickness skin graft harvested from the thigh. Wound closure was performed in layers. Postoperative three‐dimensional volume‐rendered CT demonstrated restoration of mandibular continuity with the reconstructed segment and satisfactory positioning of the reconstruction plate and fixation screws (Figure 7).

**Figure 6:**
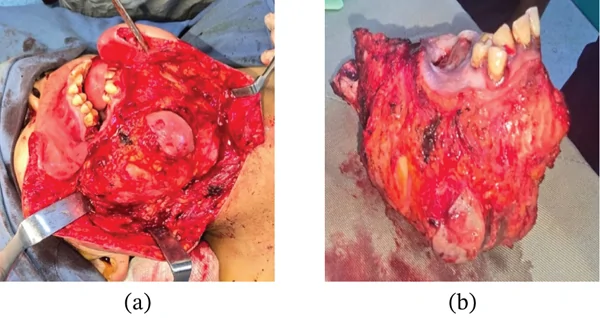
(a) Intraoperative photograph and (b) macroscopic view of the surgical resection.

**Figure 7:**
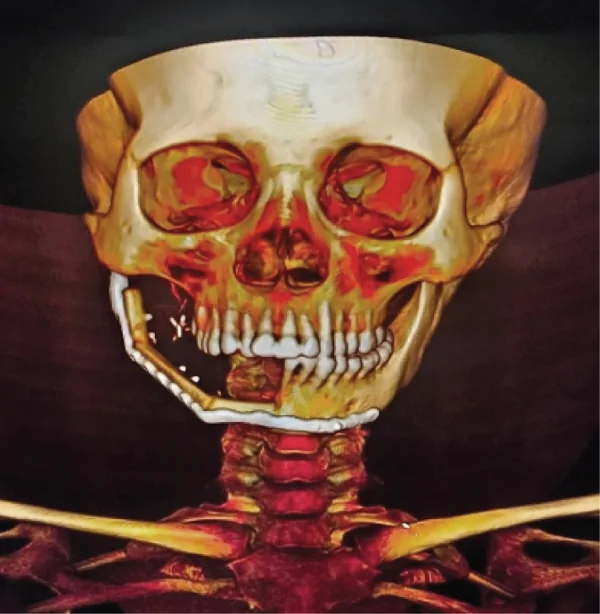
Postoperative three‐dimensional volume‐rendered CT resconstruction demonstrating restoration of mandibular continuity with resconstructed segment and satisfactory positioning of resconstruction plate and fixation screws.

Histopathological examination of mandibular resection specimen revealed involvement of the surrounding soft tissue, with extension of the tumor to the overlying skin. The surgical margins were free of tumor, with adequate clearance at the resected margins. The findings were consistent with those observed in the repeat incisional biopsy. The tumor was arranged in anastomosing islands and well‐defined nests composed of closely packed sheets of polyhedral cells. Areas of basophilic calcification (Liesegang ring) and homogenous eosinophilic material suggestive of amyloid deposition were identified. A total of 12 lymph nodes were examined, none of which showed evidence of metastatic involvement. The patient was followed postoperatively at 3, 6, 9, and 12 months and demonstrated satisfactory healing with no clinical or radiographic evidence of recurrence.

## 3. Discussion

CEOT is a rare benign epithelial odontogenic tumor, accounting for approximately 1% of all odontogenic tumors [2, 9]. This slow‐growing, locally aggressive neoplasm has intraosseous (96%) and extraosseous (4%) forms [10]. The present case is particularly significant because the lesion demonstrated a prolonged clinical course with marked progression and an intervening diagnosis of OSCC, ultimately requiring extensive surgical management.

An important consideration in interpreting the present case is its relationship to the previously published report by Baral et al. [2] The 8‐year interval between the two reports corresponds to the increase in the patient′s age. In the earlier report, the patient was 26 years old and presented with a posterior mandibular swelling without mucosal ulceration. In contrast, at 34 years of age, the patient in the present report exhibited a markedly more extensive clinical presentation, characterized by a long‐standing nonhealing ulcer, pronounced extraoral swelling with facial asymmetry, trismus, and an ulceroproliferative intraoral lesion. The marked difference in clinical presentation emphasizes the progressive and locally aggressive nature of the lesion.

The demographic characteristics of the present case are consistent with the recognized profile of CEOT, which predominantly affects adults, particularly in the third to fifth decades, with no established sex predilection [2, 11, 12]. The present patient, a 34‐year‐old woman, had a lesion involving the posterior mandible, a site commonly affected by CEOT [2, 11, 12]. However, the current presentation was notable for its extensive local progression. The lesion measured approximately 8 × 5 cm and involved the mandibular body and ramus, with cortical breach, soft‐tissue extension, and mucosal and cutaneous ulceration. In contrast to the patient′s earlier presentation, which consisted primarily of posterior mandibular swelling without mucosal ulceration [2], the current ulceroproliferative lesion closely mimicked OSCC clinically. Although CEOT is generally characterized by slow growth, local cortical destruction and extension into adjacent soft tissues have been documented [2, 8, 10, 11, 13]. Thus, the present case represents an unusually extensive and locally aggressive clinical manifestation of CEOT.

Radiographically, CEOT initially appears as a well‐defined radiolucent lesion. With progression and maturation, it gradually develops mixed radiolucent–radiopaque features due to calcification. The lesion may be either unilocular or multilocular and may exhibit a characteristic “soap‐bubble” appearance [8, 9, 11]. Radiopaque bodies within CEOT have occasionally been reported to produce a “driven snow” appearance, which may reflect the direction of tumor growth [14]. In the earlier report, the lesion demonstrated mixed radiolucent‐radiopaque features with a relatively distinct border in anterior and posterior regions and was associated with an impacted third molar [2]. In the current presentation, contrast‐enhanced CT demonstrated a large, ill‐defined lytic lesion involving the right mandibular body and ramus. The lesion demonstrated an enhancing solid component with coarse internal calcifications, cortical breach, and extension into the oral cavity, with abutment of the right lateral border of tongue. Thus, although the anatomical distribution and mixed radiographic character remained compatible with CEOT, the current imaging findings reflected a substantially more extensive lesion than that documented previously.

CEOT is diagnosed histologically by the presence of polyhedral neoplastic cells with eosinophilic, coarsely granular cytoplasm, nuclear and cellular pleomorphism, and prominent nucleoli. Most tumor cells form anastomosing sheet‐like aggregates. This tumor is characterized by extracellular eosinophilic homogeneous material resembling amyloid, as well as concentric calcific deposits known as Liesegang rings [2, 8, 11, 15]. The presented case demonstrated anastomosing islands and well‐defined nests of polyhedral cells, together with homogenous eosinophilic material suggestive of amyloid‐like deposition and basophilic calcification (Liesegang rings). These findings were consistent with the histopathological spectrum of conventional CEOT and were concordant with the findings reported by Baral et al. [2]. The intraosseous and extraosseous forms of CEOT have the same histomorphology, although the latter may contain very little or no calcified material [16]. The exact nature of the amyloid‐like material remains controversial, with studies suggesting the presence of cytokeratin, enamel‐related proteins, and basement membrane components [17]. The amyloid‐like material in CEOT is believed to induce stromal cells to produce a collagenous matrix that later mineralizes and resembles cementum. However, most of the calcified homogeneous material in the CEOT stroma is considered to represent dystrophic calcification [12, 16].

The morphologic spectrum of CEOT has also expanded over time. Previously described patterns include clear cell, Langerhans cell‐rich, cementum‐like, cystic, and mixed epithelial odontogenic tumor‐like patterns [10–12, 16, 18]. However, the 5^th^ edition of WHO Classification recognizes three histological variants: clear cell, cystic/microcystic, and noncalcifying/Langerhans cell‐rich CEOT. The latter remains controversial due to its considerable clinical and histopathological overlap with the amyloid‐rich variant of odontogenic fibroma [4, 13].

The diagnostic challenge in the present case arose largely from the extensive clinical presentation and the squamoid morphology of the tumor cells. CEOT may mimic squamous cell carcinoma histologically because of its polyhedral cells and eosinophilic cytoplasm, whereas cellular pleomorphism can further increase the resemblance to squamous malignancy [5]. Although CK5/6 and p63 are expressed in CEOT, their discriminatory value is limited because they are also commonly positive in OSCC. Ki‐67 may provide supportive information regarding proliferative activity but lacks a definitive diagnostic cutoff. Thus, differentiation relies primarily on morphology, particularly the identification of amyloid‐like material and characteristic calcifications, with Congo red staining serving as a supportive investigation when performed, together with appropriate clinicoradiographic correlation [15, 19]. Consequently, the diagnosis in the present case relied primarily on the characteristic morphological findings and their correlation with the clinical and radiographic features.

Treatment modalities for CEOT range from simple enucleation to extensive resection, including segmental or hemimandibulectomy [1–3, 11]. The choice of treatment depends on the clinical, histological, and radiographic characteristics of each case. In most nonaggressive, slow‐growing lesions, complete surgical excision with adequate tumor‐free margins is considered the treatment of choice [1]. In the present case, the markedly extensive nature of the lesion and involvement of the mandible and adjacent soft tissues necessitated wide surgical excision with an extended supraomohyoid neck dissection and subsequent free fibula flap reconstruction. No regional lymph‐node metastasis was identified, which is an important pathological finding in this extensively destructive lesion.

CEOT generally has a favorable prognosis, with recurrence occurring infrequently, whereas malignant transformation is exceptionally rare [3]. Franklin and Pindborg reported a recurrence rate ranging from 15% to 30% within 2–4 years following enucleation and curettage, whereas other studies have reported recurrence rates of approximately 10%–20% following conservative treatment [8, 11]. Because of the tumor′s slow growth and potential for recurrence, follow‐up for 5–10 years may be required [11]. In the present case, no recurrence was identified during the 12‐month postoperative follow‐up. Although this represents a satisfactory early outcome, longer surveillance remains appropriate given the recognized potential for recurrence.

## 4. Conclusion

Accurate diagnosis of extensively progressed CEOT requires close multidisciplinary correlation of clinical, radiographic, and histopathological findings. Correlation of initial and repeat biopsy findings, together with review of previous pathology, is essential to reduce the risk of misdiagnosis as OSCC. This case underscores the importance of pathology review, biopsy correlation, and multidisciplinary diagnostic assessment in guiding appropriate management of diagnostically challenging odontogenic tumors.

## 5. Limitation

Congo red staining was not performed to confirm the presence of amyloid deposition, which represents a limitation of the histopathological evaluation. Additionally, the relatively short 12‐month follow‐up period limits the assessment of long‐term clinical outcomes and the potential for late recurrence.

## Author Contributions

Manika Shrestha: conceptualization, methodology, formal analysis, resources, writing—original draft, and writing—review and editing. Prabhat Chandra Thakur: supervision, validation, resources, and data curation. Dipshikha Bajracharya: methodology, supervision, validation, and editing.

## Funding

No funding was received for this manuscript.

## Consent

Written informed consent was obtained from the patient to publish this report in accordance with the journal′s patient consent policy.

## Conflicts of Interest

The authors declare no conflicts of interest.

## Supporting information


**Supporting Information** Additional supporting information can be found online in the Supporting Information section.

## Data Availability

The authors confirm that the data supporting the findings of this study are available within the article.
